# Gastric carcinoma and renal cell carcinoma as an atypical presentation of multiple primary malignancies: a case report and review of the literature

**DOI:** 10.1186/s13256-020-02576-6

**Published:** 2020-12-02

**Authors:** J. A. Martín-Pérez, C. Torres-Silva, R. Tenorio-Arguelles, D. A. García-Corona, S. Silva-González, J. A. Dominguez-Rodriguez, I. De Alba-Cruz, J. F. Nagore-Ancona, J. A. González-Luna, K. A. López-Bochm

**Affiliations:** 1 Surgical Department, “General Ignacio Zaragoza” Regional Hospital, Ejército Constitucionalista, Chinam Pac de Juárez, Calz. Ignacio Zaragoza 1711, 09220 Mexico City, MX Mexico; 2 Oncology Surgical Department, “General Ignacio Zaragoza” Regional Hospital, Mexico City, MX Mexico; 3 Pathology Department, “General Ignacio Zaragoza” Regional Hospital, Mexico City, MX Mexico; 4 Social Services and Security Institute for the State Employees (I.S.S.S.T.E.), Mexico City, MX Mexico

**Keywords:** SPM, MPM, Synchronous, Partial gastrectomy, Radical nephrectomy, Smoking, Gastric tumor, Renal tumor, Seal ring cells, Clear cell renal carcinoma

## Abstract

**Background:**

Gastric carcinoma (GC) with second primary malignancy (SPM) is the most frequent combination within the multiple primary malignancies (MPM) group. The presentation of a GC associated with a synchronized SPM in the kidney is extremely rare and unusual. This study presents a rare case of synchronous tumors, describes the main associated risk factors, and emphasizes the need to rule out SPM.

**Main body:**

We present the case of a 63-year-old Hispanic woman with a history of smoking, weight loss, and gastrointestinal (GI) bleeding. GC was diagnosed by endoscopy, and during her workup for metastatic disease, a synchronous SPM was noted in the left kidney. The patient underwent resection of both tumors with a satisfactory postoperative course. A systematic review of the literature was performed using the Medline/PubMed, Science Direct, Scopus, and Google Scholar databases. A search of the literature yielded 13 relevant articles, in which the following main risk factors were reported: the treatment utilized, the grade and clinical stage, histopathological report, and in some cases survival. It is concluded that advanced age (> 60 years) and smoking are the main associated risk factors.

**Conclusion:**

Gastric carcinoma is the second most frequent neoplasm of the GI tract and the main neoplasm that presents a SPM. MPM screening is recommended in patients with gastric cancer. The clinical discovery of MPM of renal origin is rare and hence the importance of the current report.

## Introduction

In 1930, Warren and Gates first described the concept of multiple primary malignancies (MPN) [1]. Subsequently, the definition of a synchronous tumor was standardized, according to the time of diagnosis of the second primary malignancy (SPM), with the SPM diagnosed within a period of time no greater than 6 months after the diagnosis of the primary tumor. This definition is the one used for SPM identification in the literature review [2–7].

In the case of the presence of gastric carcinoma (GC) with SPM, the incidence is higher in Asian countries [8–13]. SPMs are mainly observed in the colon, followed by the breast and the lung; a malignancy in the kidney is rare [14–16]. The incidence of synchronous stomach-kidney tumors worldwide is around 0.13–0.42%. Up to 40% are diagnosed with SPM in the early stages and 9% are diagnosed in advanced stages [17–19].

GC, according to reviews, is the neoplasm with the highest presence of SPM [3, 18–20]. Currently, there is no standardized study protocol for the evaluation of SPMs in GC.

The objective of this study is to present a case of synchronous renal cell carcinoma in a patient with GC, as well as to describe the associated risk factors and emphasize the need to rule out the possibility of a SPM during the clinical staging of the primary malignancy.

## Case report

The patient is a 63-year-old Hispanic woman with a history of peptic ulcer disease with dyspepsia and was treated with a proton pump inhibitor (PPI). She was diagnosed with *Helicobacter pylori* infection and received a triple drug therapy. Subsequently she experienced weight loss of approximately 14 kg over 2 months (BMI = 18.4; previous weight 64 kg, BMI = 23.5), and her dyspepsia did not improve. Colonoscopy and esophagogastroduodenoscopy (EGD) were performed and revealed a 4 cm pyloric ulcer and 70% pyloric stenosis; biopsies were taken at the time of the EGD. The biopsy demonstrated adenocarcinoma in signet ring cells. She was referred to the General Ignacio Zaragoza Regional Hospital Surgery Department for further evaluation. Social history was significant for an 18-pack-year smoking history. Her family history was significant for first-degree relatives with a history of cancer: one with bladder carcinoma and the other with prostate carcinoma. Her physical examination was within normal limits. Laboratory tests demonstrated normal hemoglobin and hematocrit levels, normal liver enzymes, and normal renal function; however, urinalysis was significant for both proteinuria and hematuria, and her blood type is A positive. Carbohydrate antigen (CA)125 and CA19-9 were normal. Her chest X-ray was normal for her age. A computed tomography (CT) scan of her abdomen and pelvis showed a tumor in her left kidney of approximately 140 × 97 × 85 mm with homogeneous enhancement in its upper pole. No distant metastases were evident. Surgical intervention with an exploratory laparotomy was undertaken, where a partial gastrectomy was performed with gastrojejunal anastomosis with omega of Braun reconstruction and left radical nephrectomy. Interoperative cryosections were evaluated by pathology. She had an estimated blood loss of 850 cc, with an estimated surgical time of 210 minutes. The postsurgical course was without complications. The histopathological report describes the left kidney with a size of 100 x 80 mm with a tumor in the upper and middle pole of 70 x 50 mm. The tumor type was clear cell renal cell carcinoma with a histological grade II on the Fuhrman scale with extension beyond Gerota’s capsule. The clinical stage was T4 N0 M0. The gastric tumor with dimensions of 10 × 10 mm in the antrum and pylorus showed diffuse type adenocarcinoma with signet ring cells with little differentiated histological grade and pathological stage T2 N0 M0. The patient is currently under active surveillance with monthly monitoring by the departments of surgery and medical oncology; to date her CT scan and laboratory tests demonstrate no evidence of relapse or distant metastases.

## Materials and methods

### Inclusion criteria

This systematic review was designed according to the Preferred Reporting Items for Systematic Reviews and Meta-Analyses (PRISMA) [21].

All articles written in English and Spanish related to cases of synchronous gastric and/or renal carcinoma were included. Exclusion criteria were articles not meeting the criteria for the definition of synchronous cancer [1].

### Search strategy

The study design was developed and strictly followed by all participating authors. Identification of eligible studies was performed through a systematic literature search using Medline/PubMed, Science Direct, Scopus, and Google Scholar.

The end date for the literature search was April 1, 2020. The following keywords were used in the search algorithm: ("cancer OR tumor OR neoplasia") AND ("synchronous double primary”) AND ("Gastric OR Gastric Adenocarcinoma") AND ("renal carcinoma OR kidney carcinoma). Two independent reviewers performed the literature review. The reference lists of eligible articles were manually evaluated to detect potentially relevant articles. The selection process for this article is shown in Fig. 6.

### Systematic review and data extraction

The study design did not use an exclusion factor due to the scarcity of publications on the subject. After removing duplicates, two reviewers independently selected all abstracts and articles for the full-text review. Data analysis was extracted from full-text articles.

### Statistical analysis

The PRISMA flowchart was generated by Cochrane RevMan software version 5.2.

**Fig 1.**
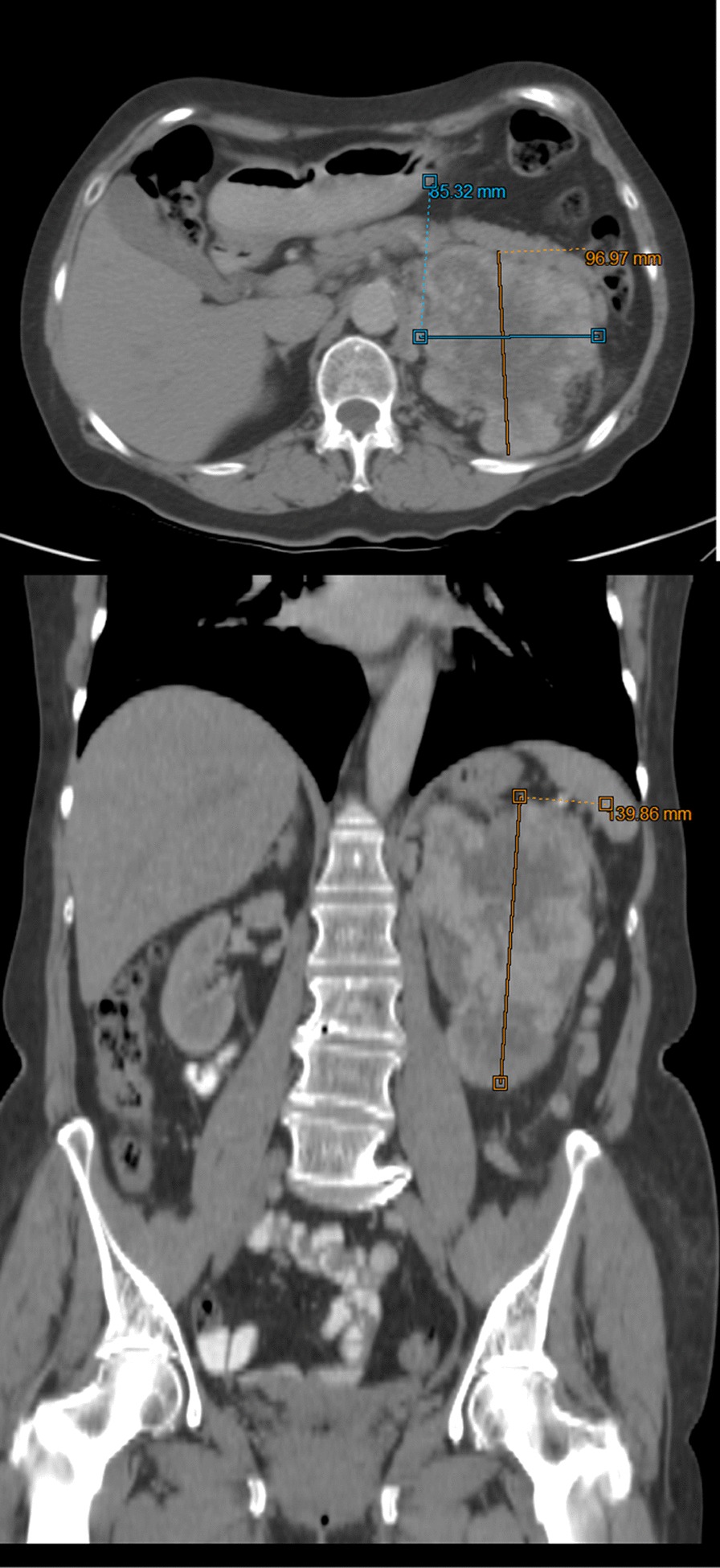
CT scan in which the left kidney cancer is observed with dimensions of 140 × 97 × 85 mm

**Fig 2.**
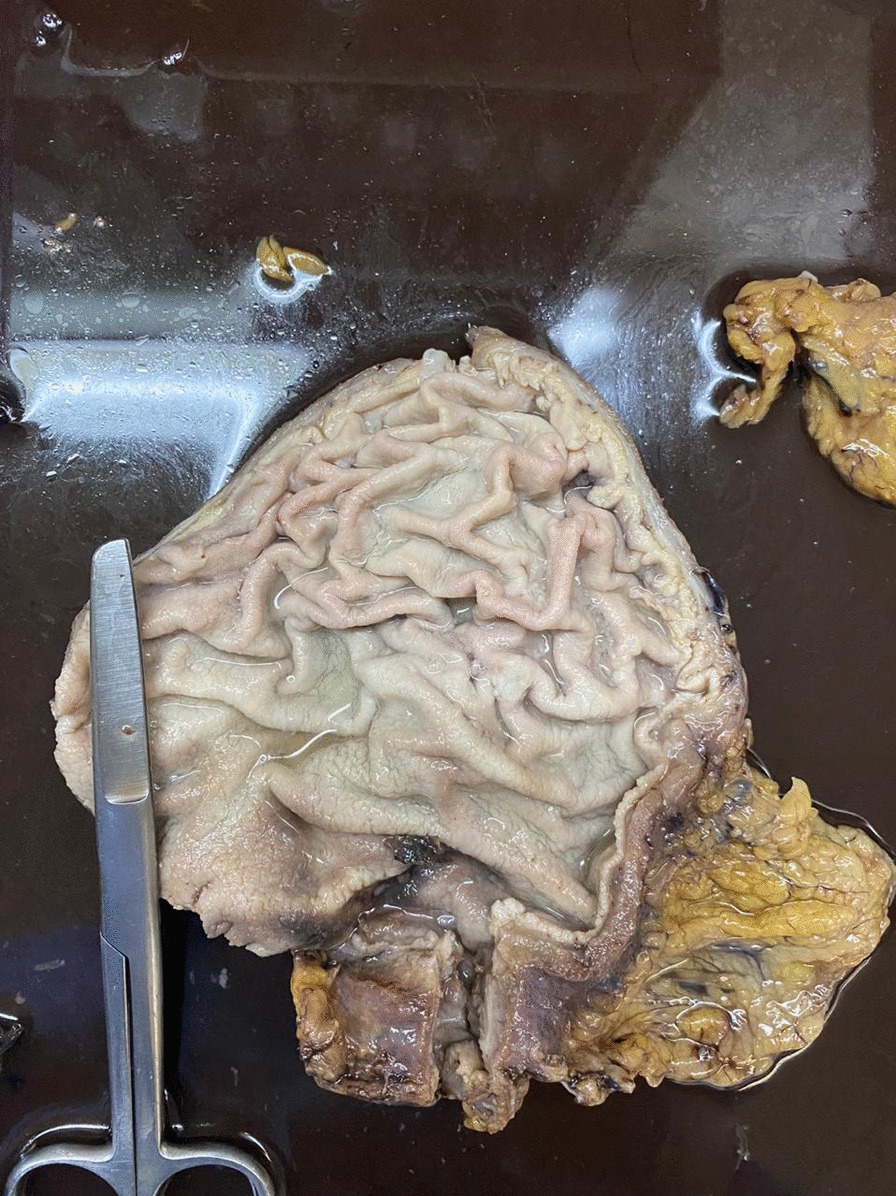
Product of partial gastrectomy (antrum and pylorus) that measures 100 × 60 × 40 mm; the cut of this area is identified

**Fig. 3:**
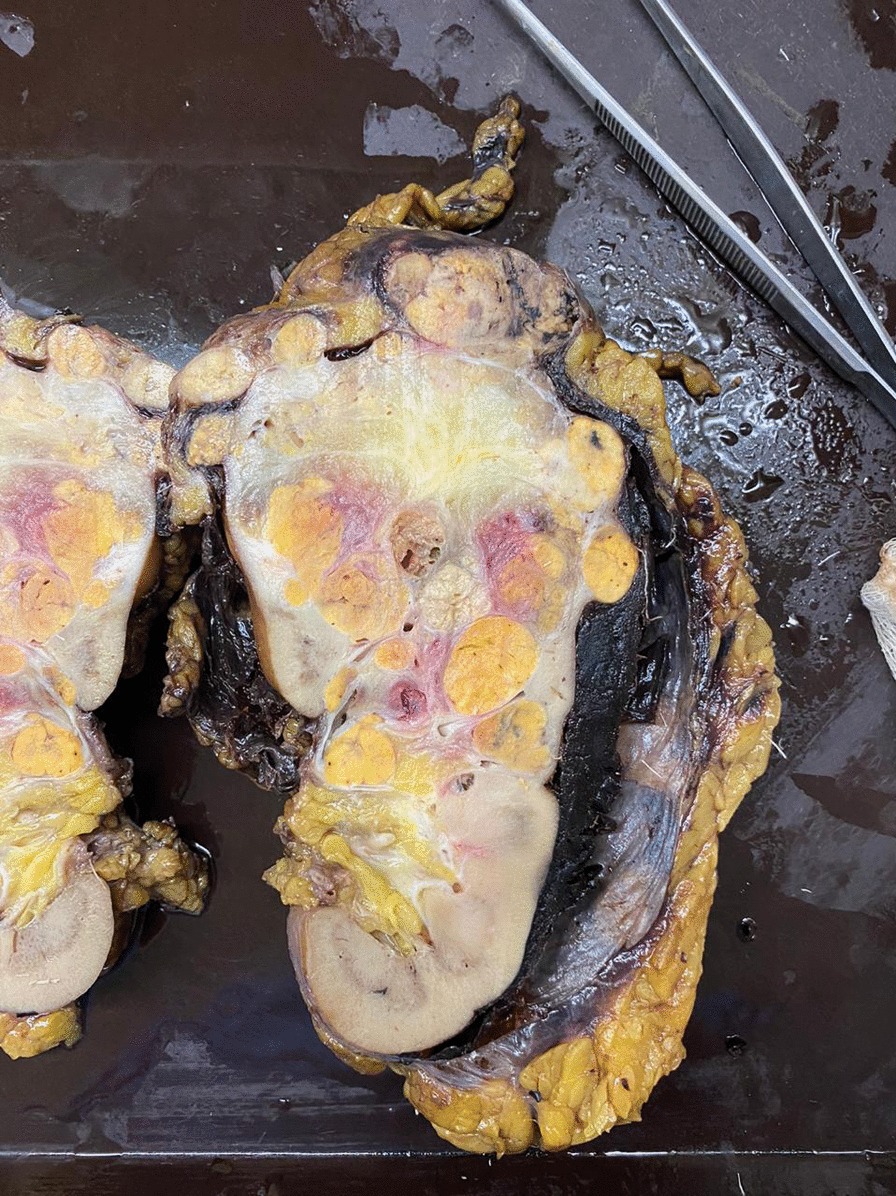
Left kidney surgical piece that measures 160 × 100 × 75 mm, in the upper pole and mid-region solid multinodular lesion

**Fig. 4:**
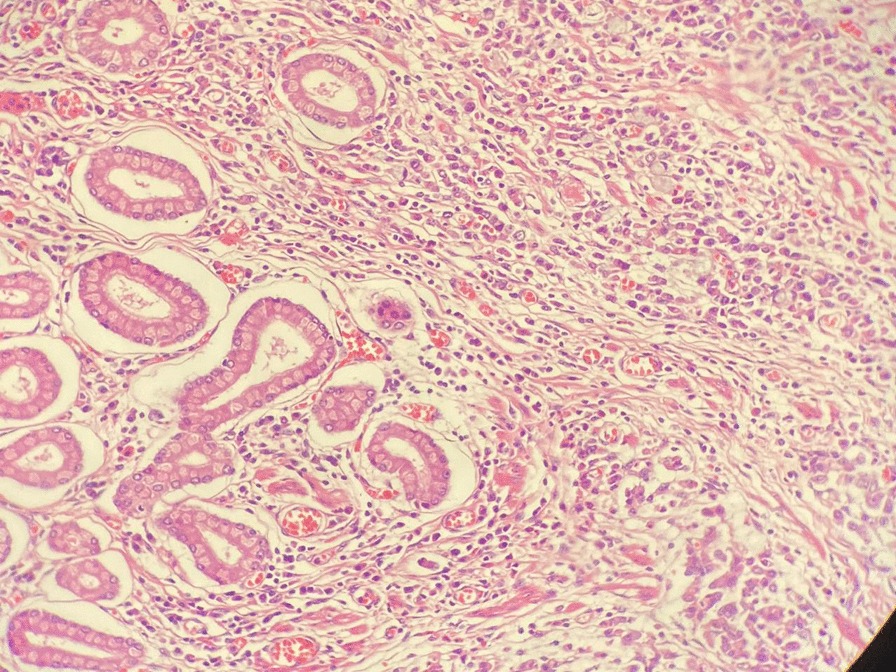
GC. Histopathological examination of antrum and pylorus (Hematoxylin and Eosin (H&E) staining x 40). In the lamina propria, individual cells of medium size are observed, with moderate pleomorphism and some in a signet ring

**Fig. 5:**
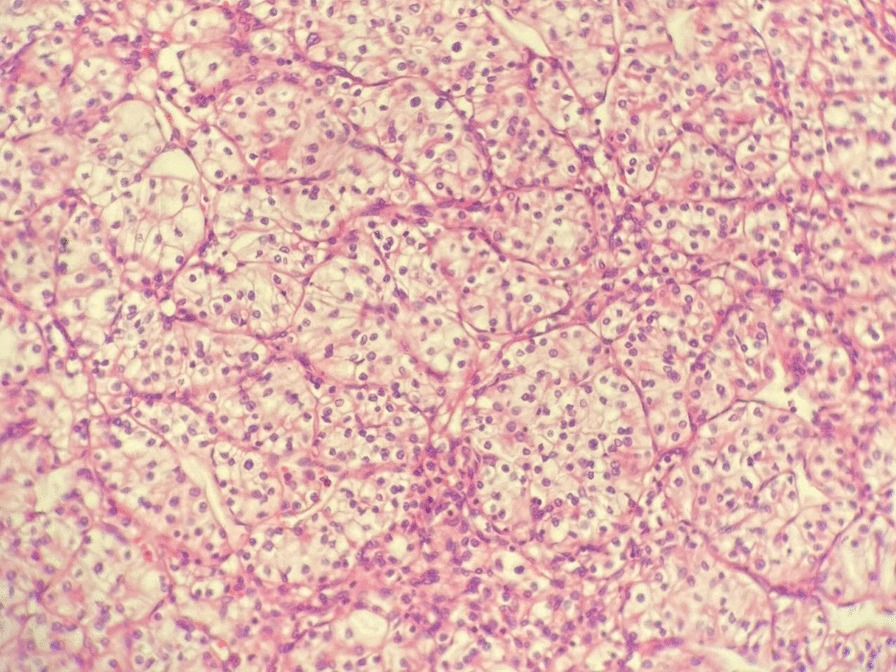
RCC. Histopathological examination of the left kidney (H&E ×20) where large cell sheets with optically clear cytoplasm and round nuclei of fine chromatin are observed

**Fig. 6:**
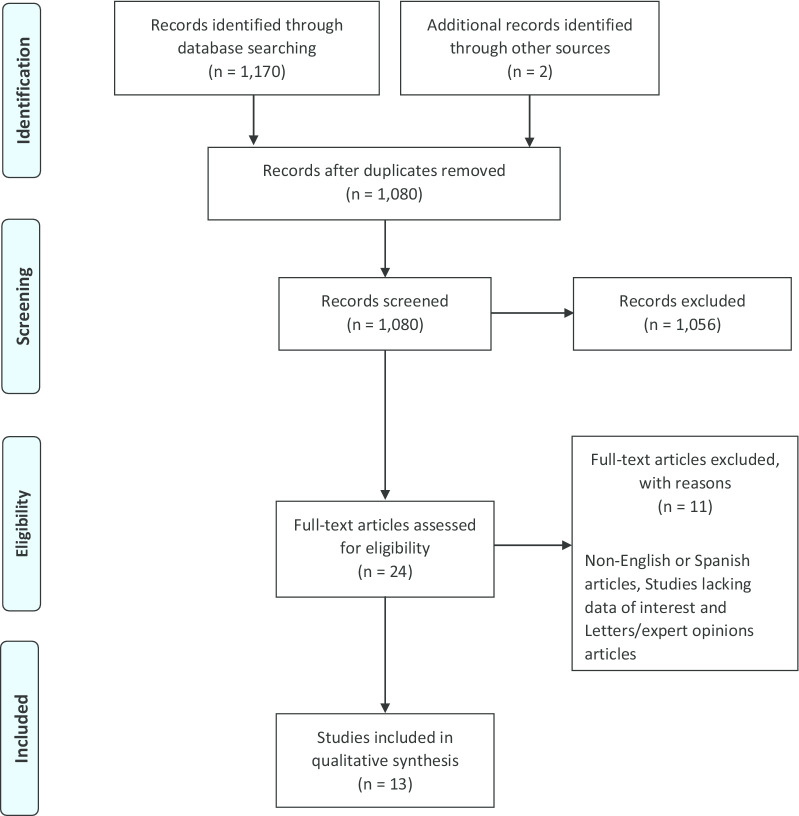
PRISMA flow diagram detailing the search and identification strategy for the studies used in data synthesis

The initial literature search identified 1172 articles. Of these, 1159 articles were excluded as they did not meet the review criteria, were duplicates, were not in the included language, were studies without data of interest, or were letters/opinions from experts. Thirteen studies were selected which met the inclusion criteria. Manual search and cross-checking of reference lists of included articles yielded no additional relevant articles. Figure 1 shows a flow diagram illustrating the identification and inclusion/exclusion processes in the study (Figs. 2, 3, 4, 5, 6).

## Results

**Fig. 7:**
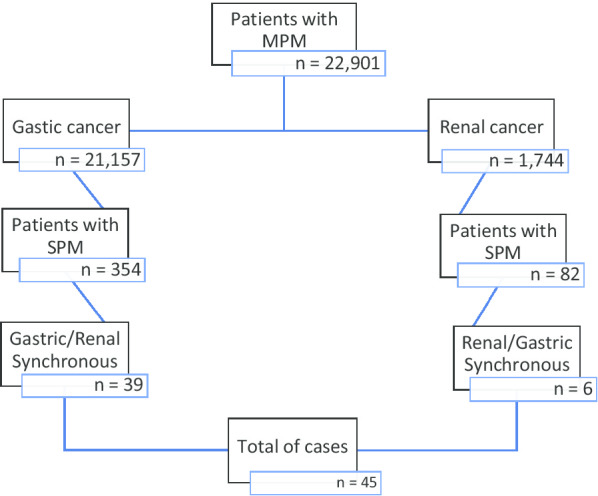
Diagram explaining our total population and the result of gastric/renal synchronous cases

The total population studied in our review yielded 21,157 patients with GC and 1744 patients with renal carcinoma. The subgroups of patients with GC and renal carcinoma presenting with a synchronous tumor numbered 354 and 82, respectively. A total of 45 cases of synchronous tumors with GC and renal carcinoma were found in our study. Figure 7 shows, schematically, the population and how it was obtained.

Within the information search, 45 patients with a diagnosis of synchronous cancer were integrated and are described in Tables 1 and 2. However, in the studies with an epidemiological design described in Table 1, it was not possible to collect particular data about the cases because they described patients with synchronous cases in general and not exclusively synchronous gastric cancer with kidney cancer.

**Table 1:** Studies with epidemiological design of reference centers

Author	Country	Type of study	Population	Synchronous number of cases	Number of synchronous renal and gastric cases
Ha *et al*. [17]	Korea	Retrospective cross-sectional	10,090	90	11
Ławniczak *et al*. [18]	Poland	Retrospective cross-sectional	862	23	1
Jiménez *et al.* [22]	Spain	Case series	25	7	1
Bang *et al.*. [2]	Korea	Retrospective cohort	4593	49	9
Lee *et al.*. [3]	Korea	Retrospective cross-sectional	3291	115	5
Kim *et al.*. [4]	Korea	Retrospective cross-sectional	41	17	3
Ikeda *et al*. [26]	Japan	Retrospective cohort	2250	48	4
Beisland *et al*. [23]*	Norway	Retrospective cohort	1425	53	2
Sato *et al.*. [24]*	Japan	Retrospective cross-sectional	319	29	4

* Kidney tumor case studies

**Table 2:** Report and integration of cases presented

Author	Country	Number of cases	Sex	Age	Presentation symptoms	Risk factors	Kidney stage/clinical stage	Stomach stage/clinical stage	Renal tumor size	Renal histology	Gastric histology	Surgical treatment
Aslam *et al.*. [5]	India	1	Male	41	Weight loss, DAP, GIB	–	–	–	108 × 65 × 10 mm	Chromophobic carcinoma	Poorly differentiated	Not a candidate for surgical treatment
Leopoldo *et al.*. [20]	Mexico	1	Male	68	DAP, dysphagia	Tobacco, alcohol, T2DM	II	IIIA	70 × 60 × 40 mm	Chromophobic carcinoma	Poorly differentiated	Gastrectomy and total nephrectomy
Hu *et al.*. [19]	Taiwan	1	Female	73	GIB	–	II	IB	120 × 90 × 120 mm	Clear cells	Moderately differentiated	Subtotal gastrectomy with total nephrectomy
Oh *et al.*. [6]	Korea	1	Male	50	Incidental finding	HBP	I	IA	19 × 15 mm	Clear cells	Poorly differentiated	Total gastrectomy with partial nephrectomy
Present case	Mexico	1	Female	63	GIB, weight loss, DAP	Tobacco, PUD	IV	IB	140 × 97 × 85 mm	Clear cells	Poorly differentiated	Partial gastrectomy with radical nephrectomy

*T2DM* type 2 diabetes mellitus, *HBP* high blood pressure, *PUD* peptic ulcer disease, *GIB* gastrointestinal bleeding, *DAP* diffuse abdominal pain

Of the total number of cases reported in the literature with synchronous GC and kidney carcinoma, the highest prevalence was found in elderly patients (> 60 years), with males outnumbering females 2:1. The most commonly reported risk factor was smoking [2, 4, 17, 18, 20, 22–25]. The most frequent clinical presentations were upper or lower gastrointestinal (GI) bleeding, nonspecific abdominal pain, weight loss without apparent cause, and absence of upper/lower urinary tract symptoms in most of the cases.

Multiple combinations of surgical treatments are described, including partial gastrectomy/radical nephrectomy [5], subtotal gastrectomy/partial nephrectomy [6, 19], total gastrectomy/radical nephrectomy [17, 20], endoscopic gastric resection/radical nephrectomy [24], partial gastrectomy/total nephrectomy [17], mucosectomy/renal active surveillance [18], and total gastrectomy/renal active surveillance [22]. Among the surgical approaches for gastric cancer, partial gastrectomy was predominant, followed by total gastrectomy and subtotal gastrectomy. Two patients were diagnosed at an early stage, and conservative endoscopic approaches were adopted for treatment. With regard to the management of patients with renal carcinoma, multiple reviews revealed that radical nephrectomy was the most frequently performed surgical intervention, followed by total nephrectomy, partial nephrectomy, and two cases treated with active surveillance. Diagnostic renal carcinoma was more frequently on the left side with nephrectomy (partial, total, or radical) [5, 6, 19, 22–24].

Our review showed the prevalence of clinical stages in GC, with stage IA being the most frequent, followed by stages IB, IIA, and IIIA [2, 6, 17, 20, 26]. On the other hand, stage II was the most frequent found for renal carcinoma [6, 20].

It was revealed that the most common histopathological findings of GC were described as poorly differentiated [2, 4, 20], moderately differentiated [19, 22], and well differentiated [17, 26], in that order of presentation. The findings in the group of patients with renal carcinoma were cell carcinomas [2, 3, 6, 19, 24] as the most frequent histopathological result, with other rare reported cases of chromophobe cell [5, 20, 23] and transitional cell carcinoma [2].

In addition, some cohort studies (presented in this review) [2–4, 17, 18, 23, 24, 26] revealed that survival and prognosis are improved in early clinical stage/grade and when both tumors were resected at the same surgical time.

The heterogeneous characteristics of the reviewed studies, the low number of relevant articles found, and the lack of specific studies in the literature limit the ability to make solid recommendations, and represent the main limitations of this systematic review.

## Discussion

Gastric carcinoma (GC) is the second most frequent GI neoplasm worldwide, occupying fifth place in cancer mortality globally and representing 4% of cancer cases diagnosed in Mexico to 2018 [27].

The incidence of GC in presentation with another SPM varies from 0.7–11% [2, 3, 5, 6, 17–20, 22, 26]. SPMs in relation to CG are most often tumors due to colorectal, lung, and liver cancer [14, 17–19, 28]. Synchronous renal cell carcinoma is very rare (0.11–0.37%) finding in our review with an incidence of 0.10% and with a 2:1 male-female ratio. The incidence of synchronous cancer has been found to be higher in GC in early stage than in advanced stages (5.2% versus 2.4%) [19, 20].

The present study describes a rare case of a female patient in her seventh decade with a history of chronic smoking, presenting with nonspecific gastrointestinal symptoms and weight loss. During the diagnostic protocol, a left kidney tumor was found. She underwent surgery by partial gastrectomy with anastomosis and left radical nephrectomy.

The most common presentation in patients seeking medical attention early is related to signs and symptoms associated with GC; the aforementioned is of utmost importance, since at the time of diagnosis they are usually in the early clinical stage, which makes it possible to carry out a comprehensive approach offering conservative and even curative treatments. However, early signs and symptoms are nonspecific, and include GI bleeding and nonspecific abdominal pain, accompanied by weight loss in up to 40% of patients over a period of 2–3 months. For this reason, it is necessary to carry out, in all cases, a workup ranging from laboratory studies (including tumor markers), radiology (abdominal CT with contrast), and EGD with biopsies to rule out or confirm the diagnosis or other incidental findings.

In our review we found that the risk factors with the greatest impact were chronic smoking and advanced age (over 60 years) [2, 4, 17, 18, 20, 22–24, 29–31]. Males are more frequently affected, with a 2:1 male/female relationship [32–35]. Another finding was that there is still no genetic alteration or association that links the synchronous presentation of both types of cancer, as noted by Betge *et al.* in their 2011 study, where they analyzed a group of patients with a well-differentiated clear cell RCC presentation and aggressive and poorly differentiated gastric cancers. They were unable to associate a known hereditary cancer syndrome, and this will have to be explored in future studies [36].

The treatment most frequently reported in our review, and which coincides with that we carried out in the present study, was the resection of both primary tumors at the same surgical time; the most commonly performed technique was partial gastrectomy with anastomosis and radical nephrectomy, since it has been shown to have a better prognosis and long-term survival in patients [37]. It is worth noting that the involvement of the left kidney (87.5%) was much more predominant than the right kidney in the articles included in our review [5, 6, 19, 22–24]. No cause or association can be concluded related to this finding.

Finally, survival is dependent on the primary tumor with the most advanced clinical stage, as this will determine the patient’s prognosis and the appropriate management, ranging from chemotherapy to radiotherapy, and even including surveillance exams to rule out a recurrence or metastasis at a distant site or the possibility of a new tumor in another organ, since these patients are predisposed to the development of MPM [38–40]. Continuing with surveillance indefinitely is suggested in this group of patients due to the risk of recurrence.

We consider that the exclusion of studies written in a language other than English or Spanish could be considered a limitation, particularly due to the high prevalence in Asia.

## Conclusion

GC is the second leading carcinoma in the gastrointestinal tract. In addition, it is the neoplasm that is associated with second primary neoplasms. It can be recommended that patients older than 60 years with a history of smoking, and especially those of the male sex, be examined for a second primary neoplasm, with the aim of early diagnosis that can increase patient survival.

## Abbreviations

GC: Gastric carcinoma
SPM: Second primary malignancy
MPN: Multiple primary malignancies
PPI: Proton pump inhibitor
EGD: Esophagogastroduodenoscopy
PRISMA: Preferred Reporting Items for Systematic Reviews and Meta-Analyses
T2DM: Type 2 diabetes mellitus
HBP: High blood pressure
PUD: Peptic ulcer disease
GB: Gastrointestinal bleeding
DAP: Diffuse abdominal pain
